# A Case of Posterior Cortical Atrophy in Alzheimer’s Disease

**DOI:** 10.7759/cureus.74403

**Published:** 2024-11-25

**Authors:** Christian Kaftanic, Neeharika Muddana, Ryan Meng

**Affiliations:** 1 Internal Medicine, Camden Clark Medical Center, Parkersburg, USA; 2 Internal Medicine, West Virginia School of Osteopathic Medicine, Lewisburg, USA

**Keywords:** alzheimer's disease, neurodegenerative disesase, neurologic processing error, posterior cortical atrophy, visual disturbance

## Abstract

This is a case of a 67-year-old male diagnosed with posterior cortical atrophy. Posterior cortical atrophy is an underdiagnosed phenomenon seen in neurodegenerative diseases, such as Alzheimer’s disease. When this condition manifests in patients with Alzheimer’s disease, it is known as a visual variant of Alzheimer’s disease. This condition leads to visual disturbances that are due to processing errors in the brain, leaving cognitive functioning unchanged. Patients tend to undergo years of unnecessary ophthalmologic workup leading to unnecessary cost and frustration to the patient. The presentation and diagnostic workup of this disease are discussed in this case report.

## Introduction

Posterior cortical atrophy (PCA) is a rare neurodegenerative syndrome characterized by progressive degeneration of the cerebral cortex, primarily affecting the occipital and parietal lobes [1]. This condition can arise from various etiologies, including Alzheimer’s disease, prion disease, corticobasal degeneration, Lewy-Body dementia, and subcortical gliosis [2]. Among these, Alzheimer’s disease is the predominant cause, accounting for approximately 96% of PCA cases [3]. A subset of Alzheimer’s patients may present with visual disturbances as the initial and chief complaint, a presentation known as the visual variant of Alzheimer’s disease (VVAD).

VVAD is notoriously difficult to diagnose due to its non-specific visual symptoms and the absence of early cognitive or memory impairment. This is attributed to the localization of amyloid plaques and neurofibrillary tangles in the parietal and occipital lobes, sparing the areas of the brain associated with memory. Common symptoms of VVAD include blurry vision, changes in color perception, homonymous hemianopia, dyslexia, or agnosia, all of which reflect disruptions in visual processing rather than primary visual defects [2].

Patients often first seek evaluation from an ophthalmologist due to the predominance of visual symptoms. However, frustration typically arises when standard ophthalmologic assessments produce normal or unremarkable results, leading to diagnostic delays. This delay can pose significant risks to patients as the disease progresses, including increased susceptibility to injuries, such as falls. This case study presents a 67-year-old male with a chief complaint of blurry vision, highlighting the clinical journey and diagnostic challenges associated with VVAD.

## Case presentation

A 67-year-old male with a past medical history of diabetes mellitus, hypertension, hyperlipidemia, and coronary artery disease presented to the ophthalmology clinic with a chief complaint of progressive blurry vision in both eyes over several years. The patient described his vision as “looking through a fog” and noted difficulties with reading road signs and distinguishing vehicles, as well as trouble recognizing facial features, which appeared blurry to him. He reported an inability to differentiate between types of vehicles, such as cars and trucks, passing by his home. The patient denied headaches, eye pain, peripheral vision changes, memory problems, or confusion and was alert and oriented to person, place, and time.

Initial visual acuity testing was notable for a significant disparity: 20/400 in the right eye and 20/30 in the left. Afferent pupillary responses were normal in both eyes, and no abnormalities were noted in color vision, disk edema, or pallor. MRI imaging performed prior to the clinic visit showed no mass effect, intracranial hemorrhage, edema, or infarcts, with normal orbital appearances. Electroretinogram results were within normal limits, and Humphrey's visual field testing revealed a minor inferior defect in the right eye. Due to the persistence of unexplained visual symptoms, the patient was referred to a neurologist for further evaluation.

**Figure 1:**
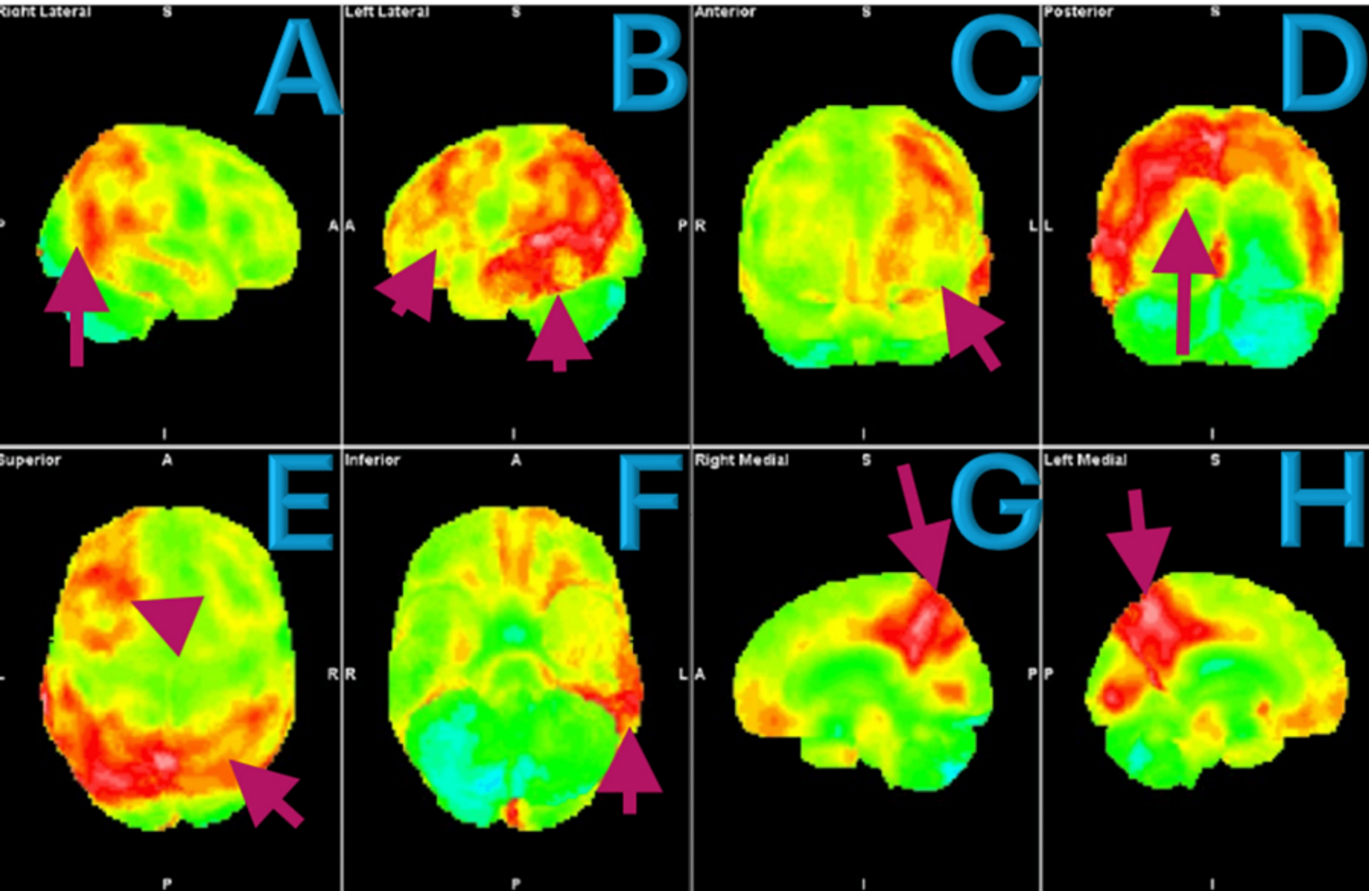
Amyloid PET-CT of the brain (A, B) Right and left lateral views of the brain, respectively. (C, D) Anterior and posterior views. (E, F) Superior and inferior views. (G, H) Right and left sagittal views. The arrows represent amyloid deposition that can be appreciated primarily in the occipital and temporal lobes. In B and E, there is some amyloid deposition localized to the frontal lobes.

**Figure 2:**
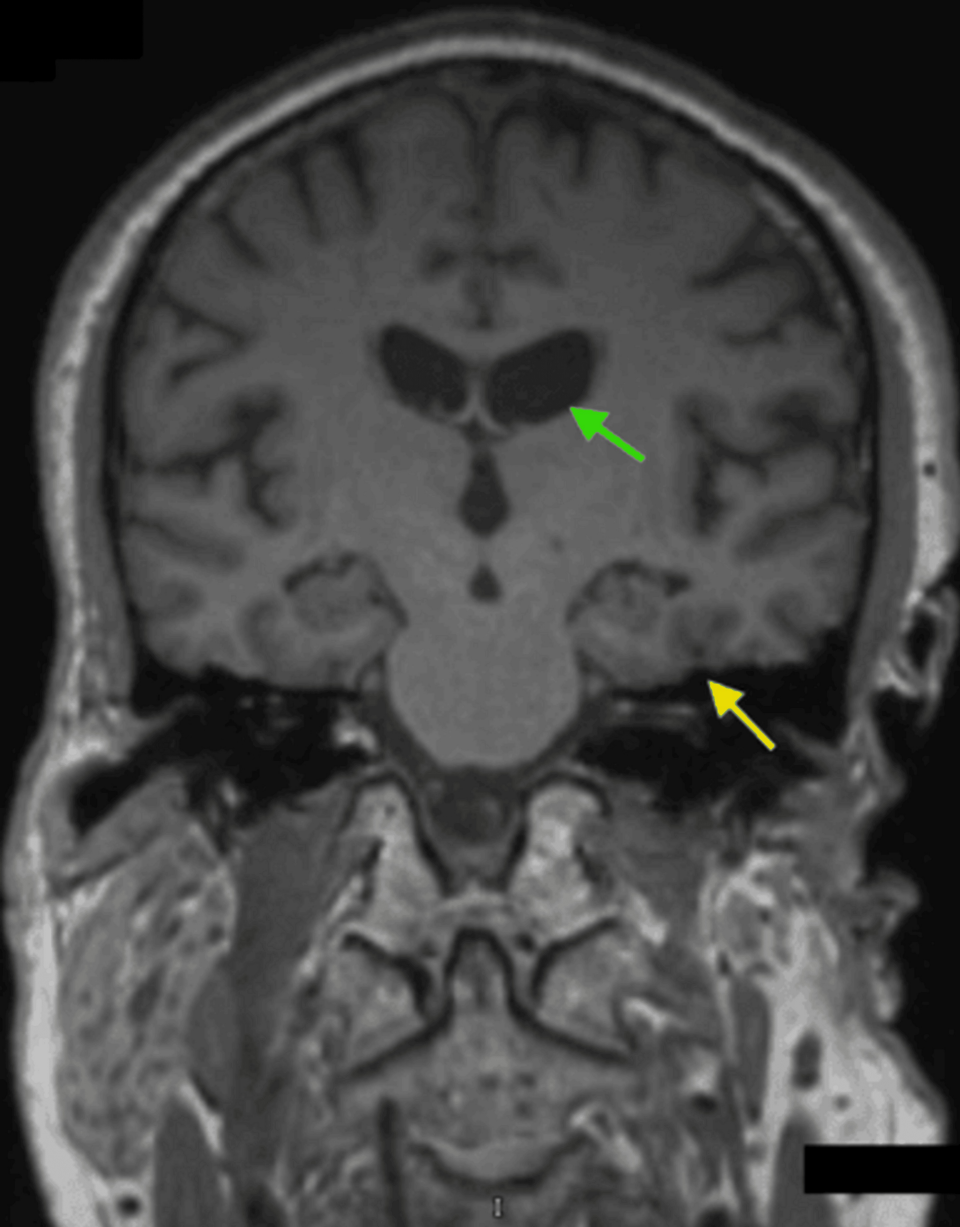
MRI of the brain demonstrating temporal lobe atrophy The green arrow is highlighting ventricular enlargement secondary to temporal lobe atrophy, which is highlighted by the yellow arrow.

The neurologist’s examination, like previous evaluations, revealed no significant neurologic deficits. The patient was appropriately oriented, with normal affect and demeanor, and displayed no physical neurologic deficits or gait abnormalities. He scored 27 on the Mini-Mental Status Exam, indicating mild cognitive impairment, and an 8 on the Geriatric Depression Scale, suggesting mild depression. Further laboratory studies, including a complete blood count (CBC), comprehensive metabolic panel (CMP), vitamin levels, and thyroid function tests, were unremarkable. A PET CT amyloid scan of the brain, as seen in Figures 1A-1H, demonstrated significant amyloid deposition in the occipital, parietal, and temporal lobes, particularly in the left occipital lobe, consistent with Alzheimer’s disease. MRI imaging showed severe temporal lobe atrophy, which can be seen in Figure 2.

## Discussion

The patient’s PET CT amyloid imaging was consistent with a diagnosis of Alzheimer’s disease, showing significant amyloid deposition in the occipital, parietal, and temporal lobes, which likely contributes to his visual disturbances. The patient exhibited key symptoms including object perception deficit, alexia, and apperceptive prosopagnosia. Visual acuity testing revealed a severe deficit in the right eye compared to the left, which may correlate with amyloid deposition in the left occipital lobe. The temporal lobe involvement might explain the patient’s difficulties with facial recognition, as processing errors in this region can disrupt face perception. Notably, the majority of the patient’s frontal cortex, except the left frontal lobe, remained unaffected. This relative sparing of the frontal regions likely accounts for the patient’s Mini Mental Status Exam score of 27, indicating preserved cognitive function without significant decline.

Posterior cortical atrophy (PCA) is often a frustrating condition for patients due to its gradual progression and the prolonged time typically required to reach a diagnosis. This delay can result in multiple referrals to specialists, along with extensive testing and procedures. Early identification of PCA can streamline the diagnostic process, reduce patient burden, and facilitate timely and appropriate treatment.

Given the broad and non-specific nature of PCA symptoms, Crutch, et al. have proposed inclusion and exclusion criteria for diagnosing PCA [4]. Inclusion criteria for early disease presentation encompass symptoms such as space perception deficit, simultanagnosia, object perception deficit, constructional dyspraxia, environmental agnosia, oculomotor apraxia, dressing apraxia, optic ataxia, alexia, left/right disorientation, acalculia, limb apraxia (excluding limb-kinetic), apperceptive prosopagnosia, agraphia, homonymous visual field defects, and finger agnosia. Crucially, higher executive functions are largely preserved, with memory, speech, personality, and behavior remaining intact. Imaging findings should include atrophy or involvement of the occipital-parietal lobes, with sparing of other brain regions. Exclusion criteria focus on ruling out alternative causes of neurological dysfunction, such as mass effects or cerebrovascular disease.

At the time of this report, the patient has not yet followed up with his neurologist to review the PET CT results, and a formal clinical diagnosis by a specialist is pending. However, the constellation of imaging findings, unremarkable ophthalmologic examination, and the specific pattern of visual disturbances-apperceptive prosopagnosia, object perception deficit, and alexia-coupled with preserved higher executive function, strongly suggest a diagnosis of PCA secondary to Alzheimer’s disease.

## Conclusions

This case highlights critical features of posterior cortical atrophy and underscores the diagnostic challenges that can lead to years of unnecessary and costly medical interventions. Understanding the pathophysiology of PCA and recognizing its clinical presentation are key to making an accurate diagnosis. This patient’s journey reflects the typical workup for many individuals with potential PCA, often involving extensive ophthalmologic evaluations and sometimes unnecessary surgical interventions, such as cataract surgeries. Early recognition of PCA is crucial for the physical and emotional well-being of patients and their families, enabling prompt and targeted management for optimal outcomes. This case underscores the challenges of diagnosing the visual variant of Alzheimer’s Disease and highlights the importance of recognizing the diverse presentations of PCA, especially in patients whose primary complaints are visual rather than cognitive.
